# Treating Chronic Pain by Modulating Phenomenological and Psychophysiological Indices of Self-Transcendence: Single-Blind Randomized Controlled Clinical Trial Protocol

**DOI:** 10.2196/82362

**Published:** 2026-04-23

**Authors:** Kennedi Childs, Carter Minnick, Geraldine Martorella, Adam W Hanley

**Affiliations:** 1 Complementary Health Innovation Lab, College of Nursing, Florida State University, 2010 Levy Ave Ste. B0217, Tallahassee, FL, United States, 1 (850) 644-3296; 2 Institute on Digital Health and Innovation, College of Nursing, Florida State University, Tallahassee, FL, United States

**Keywords:** chronic pain, mindfulness-based intervention, self-transcendence, pain, perception of pain

## Abstract

**Background:**

Chronic musculoskeletal pain (CMP) imposes a significant psychological, physical, and emotional burden on millions of adults in the United States. A challenge in treating CMP is its tendency to become enmeshed with a person’s sense of self. Mindfulness-based interventions (MBIs) offer a promising approach to help patients disentangle pain from their sense of self. However, rigorous research is needed to determine which styles of mindfulness practice most effectively promote self-transcendence, a candidate mechanism for durable pain relief.

**Objective:**

This study aims to evaluate whether adding direct pointing instruction to traditional mindful breathing enhances self-transcendence and pain relief. We will assess the effects of 2 mindfulness training protocols, mindful breathing (MB) and MB plus direct pointing (DP) instruction, on subjective and neurophysiological markers of self-transcendence and on pain-related outcomes.

**Methods:**

In this study, we will conduct a 3-arm randomized controlled trial with 173 adults with CMP, assigned to one of the following groups: (1) 4, 30-minute sessions of traditional mindful breathing instruction, (2) 4, 30-minute sessions of MB+DP instruction, or (3) a waitlist control group. Self-transcendence will be assessed during the first and final intervention sessions using validated self-report measures and psychophysiological indices, including electroencephalography (EEG; theta activity) and functional near-infrared spectroscopy (fNIRS; default mode network [DMN] activity). Primary outcomes include changes in self-transcendence, theta activity, and DMN activity. Additional outcomes include acute pain intensity and pain-related functional interference, measured at baseline through 3-month follow-up.

**Results:**

Data collection began in October 2024 and will conclude by May 2027. As this is a protocol paper, no results are yet available. We will report recruitment rates, baseline characteristics, and initial outcome trends in the full trial results paper.

**Conclusions:**

This will be the first randomized controlled trial to examine whether direct pointing instruction enhances analgesia by eliciting self-transcendence. Findings may advance understanding of the mechanisms underlying mindfulness-based pain relief and inform the development of more targeted MBIs for chronic pain. Results will be disseminated through peer-reviewed publications, conference presentations, and stakeholder engagement, with potential implications for clinical practices.

## Introduction

### Background

Chronic musculoskeletal pain (CMP) is among the most prevalent and disabling health conditions worldwide, affecting up to one-third of the global population and significantly diminishing quality of life and workplace productivity [1-3]. In the United States, CMP is a major contributor to health care expenditures and long-term disability [1,2,4]. Given the well-documented risks associated with pharmacologic treatments, such as gastrointestinal complications from nonsteroidal anti-inflammatory drugs and the potential for addiction and overdose with opioid analgesics, guidelines from the American College of Physicians recommend nonpharmacologic treatments, such as massage, acupuncture, and mindfulness, as preferred first-line therapies for managing CMP [5]. Nonpharmacologic approaches not only offer a safer alternative to pharmacologic treatment but also more directly address the complex biopsychosocial factors that contribute to chronic pain [6]. Among the notable psychological consequences of chronic pain is its potential to alter an individual’s sense of self [7-9]. Persistent pain may become enmeshed with personal identity through repeated and simultaneous activation of self- and pain-related schemas (eg, “I am [ie, self-schema] in pain [ie, pain schema]”) [7]. Over time, this repeated activation leads to a pain-laden self-concept [8,10,11] which may be reflected in corresponding changes within the brain’s default mode network (DMN) [12-14]. The DMN is a system involved in both self-representation and pain processing, potentially trapping individuals in a self-perpetuating “pain perception box” [7,10,11].

Mindfulness-based interventions (MBIs) offer a promising means of disentangling chronic pain from the sense of self, thereby disrupting the maladaptive cycle that sustains the pain individuals endure [9]. Rather than providing mere distraction or placebo effects, mindfulness engages specific neural and psychological mechanisms that fundamentally alter pain perception and appraisal [15-17]. Central to this process is a reduction in self-referential processing, a shift supported by neuroimaging evidence showing decoupling between the thalamus and DMN hubs such as the precuneus and medial prefrontal cortex during mindfulness practice [9,15,18,19]. This decoupling may represent a loosening of the neural circuits that enmesh pain in the sense of self, facilitating a more open, present-centered awareness [9,15,18,19]. The Mindfulness-to-Meaning Theory deepens this perspective by suggesting that mindfulness initiates a recursive cycle of positive psychological processes, including decentering, attentional broadening, cognitive reappraisal, and savoring, that synergistically promote self-transcendent states [9]. These transient states, characterized by diminished self-salience and expansive positive affect (eg, awe, bliss, and compassion), weaken the cognitive and emotional enmeshment between the individual and their pain experience [9,20]. Through momentary dissolution of the subject-object dichotomy, self-transcendence can reduce the centrality of pain in consciousness and cultivate a renewed sense of meaning [9,20]. In this way, mindfulness-induced self-transcendence may not only reshape the brain’s functional architecture but may also reframe the lived experience of pain, offering a path toward relief that is both neurologically grounded and psychologically liberating [9].

Emerging empirical evidence supports this theoretical framework. Two randomized controlled trials (RCTs) have examined the therapeutic value of self-transcendent states elicited during brief (ie, 15-20-minute), single-session mindfulness-based interventions (MBIs) for adults undergoing knee or hip replacement surgery (n=196, n=118) [21]. In both RCTs, the MBIs incorporated direct pointing (DP), an ancient yet underexplored contemplative technique designed to evoke self-transcendence by guiding attention away from self-specifying information and toward the nature of awareness itself [22-24]. Specifically, DP instruction encourages deidentification with thoughts, emotions, sensations, and sensory input as a means of accessing a nondual form of awareness in which the usual subject-object distinction dissolves [22-24]. Across both RCTs, groups involving mindfulness paired with DP consistently evoked the highest levels of self-transcendence relative to the psychoeducation control group, which in turn predicted immediate pain relief and improved postoperative outcomes, including reduced pain intensity and interference during the first month after surgery and better physical functioning 6 weeks after surgery [21,25,26]. These findings highlight the potential of DP to enhance the analgesic effects of brief MBIs by targeting self-related mechanisms of pain. However, because all mindfulness groups in these RCTs included DP instruction, the specific contribution of this technique remains to be isolated.

The therapeutic value of self-transcendence has also been examined in 4 RCTs evaluating the 8-week Mindfulness-Oriented Recovery Enhancement (MORE) program as a treatment for adults with chronic pain receiving long-term opioid therapy (n=95, n=187, n=62, and n=165) [27-30]. MORE incorporates DP throughout its protocol and has been shown to increase self-transcendent experiences both in daily life [27,30] and during lab-based, self-guided mindfulness practice [28,29]. In these RCTs, self-transcendent experiences were associated with reductions in pain, opioid misuse, and opioid dosing out to 9-month follow-up, as well as increases in frontal midline theta activity, which is thought to be a neurophysiological marker of meditative absorption and self-dissolution [27-30]. Collectively, these findings suggest that self-transcendence, along with its neurophysiological correlates, may serve as a core mechanism through which mindfulness improves chronic pain-related outcomes. Yet, due to the multimodal nature of MORE, the specific effects of DP remain confounded and warrant further investigation.

### Aims

To isolate the specific effect of DP to mindfulness-based pain relief, the Pointing Beyond project will randomize 173 adults with CMP into one of three groups: (1) 4 weekly 30-minute sessions of traditional mindful breathing (MB) instruction, (2) 4 weekly 30-minute sessions of MB combined with DP (MB+ DP) instruction, or (3) a waitlist control group (WLC). The specific aims are:

#### Aim 1

Evaluate between-group differences in changes in self-transcendence from Session 1 to Session 4 across the MB, MB+DP, and WLC groups, using a validated self-report instrument (state version of the Nondual Awareness Dimensional Assessment [NADA-S]; primary outcome) and 2 psychophysiological indices (secondary outcomes), including frontal midline theta activity (via electroencephalography [EEG]) [28,29] and a functional near-infrared spectroscopy (fNIRS) [31] derived proxy default mode network (DMN) activity.

#### Aim 2

Examine the associations among phenomenological and psychophysiological indices of self-transcendence by testing within- and between-session correlations between self-reported self-transcendent state (NADA-S), frontal midline theta activity (EEG), and fNIRS-derived proxy DMN activity across intervention sessions and study groups.

#### Aim 3

Determine whether increases in self-transcendence mediate treatment-related improvements in pain outcomes by testing indirect effects of group assignment (MB, MB+DP, and WLC) on (1) acute pain intensity at the end of Session 4 (secondary outcome) [32] and (2) pain-related outcomes (eg, functional interference) through 3-month follow-up (tertiary outcomes) [33-38], via changes in (1) self-reported self-transcendence (NADA-S), (2) frontal midline theta activity (EEG), and (3) fNIRS-derived DMN activity measured during Session 4, controlling for presession pain and baseline levels of the mediators.

### Hypotheses

The following are our hypotheses:

#### Aim 1

We hypothesize that, from Session 1 to Session 4, both mindfulness groups (MB and MB+DP) will show greater increases in self-transcendence, indexed by higher NADA-S scores and greater frontal midline theta activity (EEG), and greater decreases in DMN activity, indexed by reduced fNIRS-derived fronto-temporal-parietal connectivity, compared to WLC group. We further hypothesize that MB+DP will produce larger increases in these phenomenological and neurophysiological indices of self-transcendence, and larger reductions in DMN activity, relative to MB alone.

#### Aim 2

We hypothesize that phenomenological and psychophysiological indices of self-transcendence will be significantly and coherently related across sessions and groups, such that a higher self-reported self-transcendent state (NADA-S) will be associated with greater frontal midline theta activity (EEG) and lower fNIRS-derived proxy DMN activity within sessions and across Sessions 1-4. We further hypothesize that these associations will be the strongest in the MB+DP group, intermediate in the MB group, and the weakest or absent in the WLC group, reflecting graded coupling between subjective experience and neural indices of self-transcendence as a function of mindfulness instruction type.

#### Aim 3

We hypothesize that treatment-related improvements in pain outcomes will be mediated by increases in self-transcendence and its psychophysiological correlates. Specifically, we expect that assignment to the mindfulness groups, particularly MB+DP, will be associated with lower acute pain intensity at the end of Session 4 and reduced pain-related functional interference through 3-month follow-up via (1) increases in self-reported self-transcendent state (NADA-S), (2) increases in frontal midline theta activity (EEG), and (3) reductions in fNIRS-derived proxy DMN activity measured during Session 4, controlling for presession pain and baseline levels of each mediator [39,40].

## Methods

### Overview

**Figure 1.**
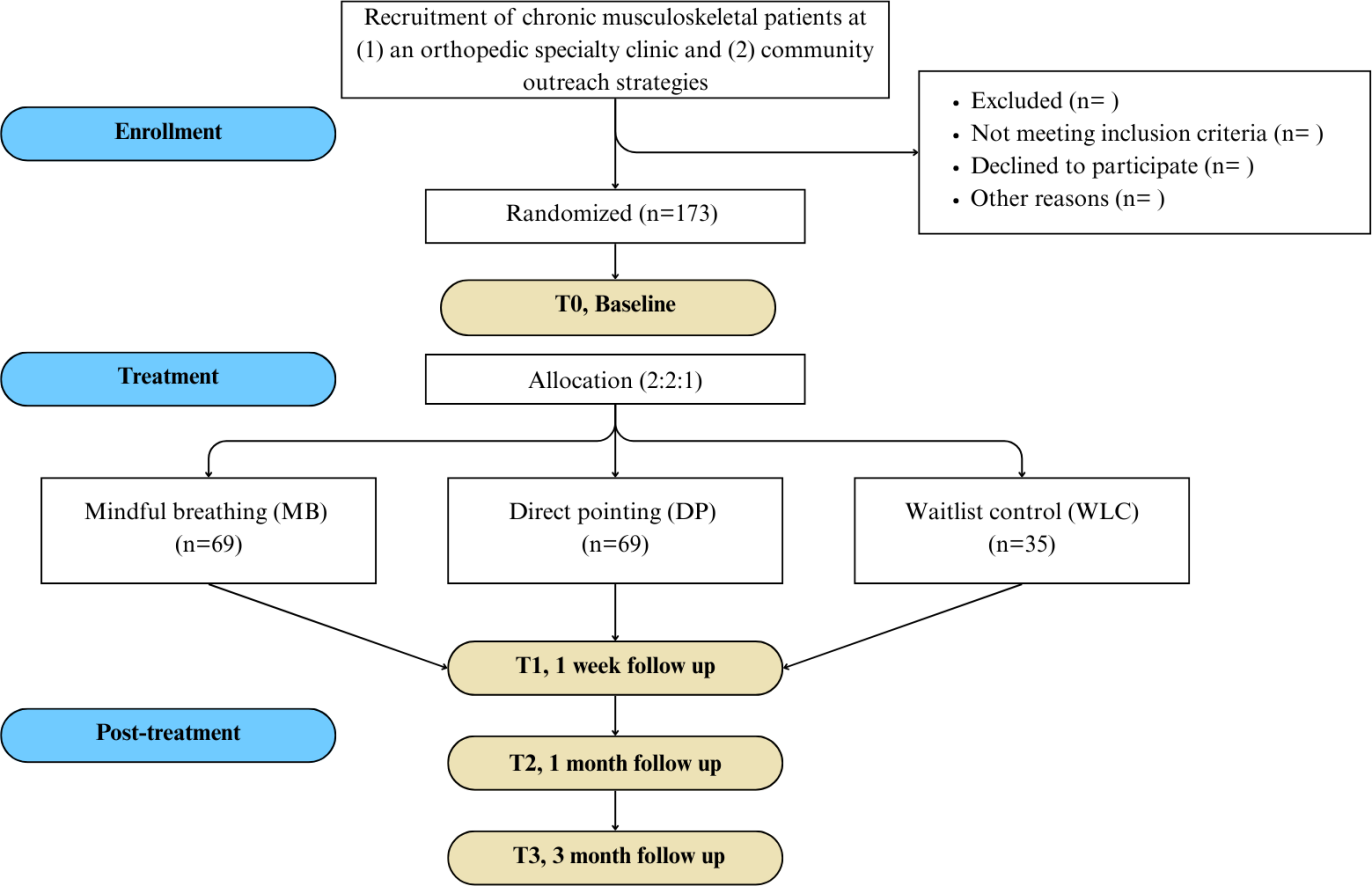
Diagram of planned study flow. DP: direct pointing; MB: mindful breathing; WLC: waitlist control.

This will be a single-blind, randomized, 3-arm, parallel-group, clinical trial (2:2:1 allocation ratio). The planned flow diagram for this single-site RCT is presented in Figure 1. The protocol is reported according to the SPIRIT (Standard Protocol Items: Recommendations for Interventional Trials; Checklist 1) [41] checklist. This study was prospectively registered with the Open Science Framework on April 25, 2023.

### Participants

We will recruit and enroll 173 adults with CMP who meet established eligibility criteria. Sample size estimation was conducted in 2 stages, reflecting an expansion from a sole focus on mechanistic sensitivity to the joint consideration of mechanistic and clinical relevance. The first stage was designed to detect changes in self-transcendence in order to elucidate an emerging therapeutic mechanism of MBIs, whereas the second stage broadened the scope of the study to contribute to a growing body of research evaluating the effects of brief MBIs on chronic pain.

First, our original power analysis using G*Power (Heinrich Heine University Düsseldorf) indicated that a total sample of 138 participants would provide sufficient power (*β*=.80) to detect changes in self-reported and psychophysiological indices of self-transcendence [42]. This target reflected a required analyzable sample of 110, inflated by 25% to account for expected attrition. To promote robust detection of mechanistic effects, we specified a conservative effect size ( Cohen *d*=0.30), representing half the smallest effect observed in our preliminary data (Cohen *d*=0.60). This approach was intended to support the more stringent comparison between the MB and MB+DP groups, while incorporating corrections for multiple comparisons.

Second, to enhance the clinical interpretability of the intervention effects, we added a waitlist control group. This adjustment was made prior to participant enrollment. Inclusion of this no-treatment comparison group provides a benchmark against which to evaluate the specific impact of the MBIs on both mechanistic and clinical outcomes, relative to the natural progression of symptoms in the absence of additional intervention. This control arm was designed to be half the size of each active treatment group (n=35), resulting in a total sample size of 173 and preserving a 2:2:1 randomization ratio. This total sample size (n=173) is consistent with a G*Power estimate for a 3 (group)×2 (time) mixed design, assuming a small effect (Cohen *d*=0.30), 80% power, and a Bonferroni-adjusted alpha of .0125 for 4 pairwise comparisons (ie, self-reported self-transcendence; 2 psychophysiological indices of self-transcendence, including frontal midline theta activity and DMN activity, and acute pain). Beyond these primary group-level effects, the planned sample size also supports Aims 2 and 3. Specifically, a G*Power analysis (2-tailed, *α*=.0167, corrected for multiple comparisons; power=0.80) indicated that 112 participants are sufficient to detect a moderate association (*r*=0.30) among phenomenological and psychophysiological indices of self-transcendence, well below the planned sample of n=173. Finally, this sample size is adequate for the planned mediation analyses; the parallel multiple-mediator model for acute pain includes 19 estimated parameters, yielding approximately 9.1 observations per parameter with n=173, consistent with the commonly cited 5‐10 observations per parameter convention.

### Eligibility Criteria

Participants will be eligible for inclusion in the study if they meet the following criteria (Textbox 1):

Textbox 1. **Participant inclusion and exclusion criteria.**

The following criteria were used to determine participant eligibility for the study.

**Inclusion criteria**

- Age 18 years or older
- Diagnosis of chronic musculoskeletal pain (CMP) by a licensed medical provider
- Self-reported average pain intensity of ≥3 on a 0–10 numeric rating scale, experienced on most days over the past 3 months
- Ability to understand spoken and written English
- Ability to complete online self-report questionnaires using a tablet or personal computer

**Exclusion criteria**

- Inability to provide informed consent due to cognitive, psychiatric, or physical impairment
- Previous formal mindfulness training (eg, completion of Mindfulness-Based Stress Reduction or equivalent structured programs)
- Diagnosis of cancer at the time of enrollment
- Active psychosis, suicidal ideation with intent or plan, or severe substance use disorder, as determined by a structured clinical interview (Mini-International Neuropsychiatric Interview [MINI])
- Presence of an unstable medical condition that could interfere with study participation or safety
- Surgery within the past 3 months
- Receipt of pain-relieving injections (eg, corticosteroid or hyaluronic acid) within the past 3 months

Relevant concomitant care is restricted during the trial. Patients are welcome to continue their usual medical care but are asked to refrain from beginning any new treatments during the study. Cortisone and hyaluronate injections are prohibited during the trial.

### Study Settings

All mindfulness training sessions and psychophysiological assessments will be conducted at the Complementary Health Innovation Lab (CHIL), located within Florida State University’s (FSU) College of Nursing. CHIL is equipped with a dedicated psychophysiology laboratory that integrates EEG and fNIRS systems, allowing for simultaneous data acquisition via a unified interface.

Sessions involving psychophysiological data collection (Sessions 1 and 4) will be conducted in a low-light environment to optimize the quality of fNIRS signal acquisition and more closely approximate real-world mindfulness settings. During these sessions, the participant, study therapist, and trained research assistant (RA) will be present. For Sessions 2 and 3, which do not involve psychophysiological data collection, only the participant and the therapist will be in the lab room. The treatment environment remains identical across all sessions, with the sole distinction being that the simultaneous EEG and fNIRS recording cap is worn only during the psychophysiology sessions.

Self-report measures will be administered during sessions, including assessments of self-transcendence and acute pain. All in-session measures will be completed using a tablet-based survey via the Qualtrics (Qualtrics International Inc) platform. Broader clinical assessments, including baseline and follow-up surveys at 1 week, 1 month, and 3 months, will be conducted remotely through Qualtrics at a time and location convenient for each participant.

### Assignment of Interventions and Blinding

Participants will be randomly assigned to intervention groups using a computer-generated randomization sequence created in Microsoft Excel. Randomization is stratified by opioid use (yes or no) to ensure balance across treatment arms on this clinically relevant variable. Within each stratum, participants are allocated using permuted block randomization with a fixed block size of 6 to maintain allocation concealment and prevent prediction of assignment. Treatment allocation is generated by personnel uninvolved in assessment or treatment and concealed on the private, password-protected computer of the uninvolved research member.

To preserve blinding, the randomization sequence will not be accessible to either assessment staff or the principal investigator (PI) until the study’s completion. Treatment allocation, for active treatment groups, will only be revealed to the interventionist immediately before the participant’s first treatment appointment. Outcome assessor blinding will further be ensured through the use of online, asynchronous self-report surveys, preventing any direct interaction between study personnel and participants.

Unblinding will only occur in exceptional circumstances where knowledge of the assigned intervention is essential for the clinical safety of the participant or to address a serious adverse event (AE). The request for unblinding must be made by the PI or a designated senior clinical investigator after determining that unblinding is necessary for participant safety or ethical oversight. The PI will document the rationale for unblinding and notify the institutional review board (IRB) if the event involves a serious adverse reaction or protocol deviation that may impact participant safety or data integrity.

### Recruitment Plan

Participants will be recruited through a combination of clinical and community-based outreach strategies to ensure a diverse sample of adults with CMP. Primary recruitment will occur through an orthopedic specialty clinic located in the Southeastern United States. Potential participants will be identified through direct referrals from clinicians or may self-refer after viewing recruitment materials displayed in the clinic. To supplement clinic-based recruitment, we will implement community outreach strategies designed to engage individuals with CMP who may not be actively seeking specialty care. These strategies will include the distribution of digital and print advertisements across local community centers and libraries. Targeted social media campaigns (eg, Facebook and Instagram [Meta Platforms]) will further expand recruitment reach. All advertisements will include a brief study description, eligibility criteria, and a link to a secure online screening form. Interested individuals will be directed to complete a brief web-based prescreening questionnaire to determine initial eligibility. Those who appear eligible will be contacted by study staff for a structured telephone interview to confirm inclusion and exclusion criteria.

### AE Reporting and Harms

Participant safety and data integrity will be monitored on an ongoing basis by the PI and designated study team members. Any reported events, whether physical, psychological, or behavioral, will be documented in a standardized case report form and evaluated for severity, duration, and potential relationship to the study intervention. The PI will assess each AE for relatedness and seriousness and determine whether the event qualifies as an unanticipated problem involving risk to participants.

Any AE or unanticipated problem involving risk to participants will be reported in accordance with the local IRB policies and applicable regulatory requirements. Nonserious but study-related AEs will be recorded and reviewed regularly by the study team to identify any emerging patterns that could indicate risks associated with the intervention or trial procedures. Participants who experience distress or negative effects related to the intervention (eg, heightened anxiety) will be provided with appropriate support. All documentation of AEs will be maintained in a secure, confidential manner and reviewed periodically to ensure prompt and appropriate response.

### Patient and Public Involvement Statement

Patients and the public were not involved in the design, conduct, reporting, or dissemination plans of this research.

### Interventions

Participants in the 2 active treatment arms (MB and MB+DP) will attend 4 weekly, 30-minute, manualized mindfulness training sessions. Each session will be delivered in person by a PhD-level interventionist. In addition to the in-session mindfulness practice, all participants will be assigned daily at-home mindfulness practice throughout the 4-week intervention period. The at-home mindfulness practice will be a 15-minute, audio-recorded mindfulness practice recorded by a clinician and sent to participants via email. Each participant in the active treatment groups will receive a 15-minute audio recording that corresponds to the specific mindfulness practice style of the group to which they were randomized.

Each session will follow a consistent structure. The first session will begin with a 5-minute introduction to the intervention. In Sessions 2 through 4, the initial 5 minutes will be used to review participants’ experiences with their at-home mindfulness practice. All sessions will then include a 15-minute guided mindfulness practice and conclude with 10 minutes of therapist-led inquiry and reflective discussion. This structure is informed by established and empirically supported brief mindfulness intervention models [43]. To ensure treatment fidelity, all sessions will be audio-recorded. A study supervisor will review session recordings and provide regular supervision, ensuring adherence to the treatment manual and consistency in intervention delivery across participants. The specific elements of each session in both programs are described in Table 1.

**Table 1.** Content and practice overview of each session of mindful breathing (MB) and mindful breathing+direct pointing (MB+DP) active treatment groups.

Program session	MB^a^	MB+DP^b^	Total session duration
1	Brief introduction and pain psychoeducation (5 minutes) Formal practices: Mindfulness of breath (15 minutes) Therapeutic processing (10 minutes)	Brief introduction and pain psychoeducation (5 minutes) Formal practices: Mindfulness of breath (10 minutes) 5 minutes of direct pointing instruction Therapeutic processing (10 minutes)	30 minutes
2	Brief welcome + review of at-home practice (5 minutes) Formal practices: Mindfulness of breath (15 minutes) Therapeutic processing (10 minutes)	Brief welcome + review of at-home practice (5 minutes) Formal practices: Mindfulness of breath (10 minutes) 5 minutes of direct pointing instruction Therapeutic processing (10 minutes)	30 minutes
3	Brief welcome + review of at-home practice (5 minutes) Formal practices: Mindfulness of breath (15 minutes) Therapeutic processing (10 minutes)	Brief welcome + review of at-home practice (5 minutes) Formal practices: Mindfulness of breath (10 minutes) 5 minutes of direct pointing instruction Therapeutic processing (10 minutes)	30 minutes
4	Brief welcome + review of at-home practice (5 minutes) Formal practices: Mindfulness of breath (15 minutes) Therapeutic processing (10 minutes)	Brief welcome + review of at-home practice (5 minutes) Formal practices: Mindfulness of breath (10 minutes) 5 minutes of direct pointing instruction Therapeutic processing (10 minutes)	30 minutes

a MB: mindful breathing.

b MB+DP: mindful breathing+direct pointing.

#### Mindful Breathing (MB)

Participants assigned to the MB group will engage in a focused attention mindfulness practice adapted from validated brief mindfulness protocols [43,44]. During the 15-minute guided practice, participants will be instructed to maintain attention on the breath, particularly sensations at the nostrils and abdomen, and to gently return their focus to the breath when distracted by thoughts, emotions, or bodily sensations. When pain arises, they will be guided to acknowledge it nonjudgmentally, without resistance or elaboration, before returning their attention to the breath.

#### Mindful Breathing+Direct Pointing (MB+DP)

Participants assigned to the MB+DP group will receive the same initial instructions as the MB group for the first 10 minutes of practice. During this time, foundational mindfulness skills are cultivated to support receptivity to the DP instruction. In the final 5 minutes of the guided practice, the therapist will deliver DP instructions, an introspective, contemplative technique derived from Mahamudra and Dzogchen traditions [22,23]. DP aims to shift attention from the contents of awareness (eg, pain and thoughts) to the nature of awareness itself [22,23]. Instructions will guide participants to experientially investigate the spacious, nonconceptual qualities of awareness, often described in classical sources as possessing inherent clarity and bliss [22,23]. The DP phase is designed to induce a transient, self-transcendent state in which rigid identification with self-referential content (eg, “I am in pain”) may dissolve.

#### Waitlist Control (WLC)

The WLC group was included to provide a reference for the natural progression of symptoms in the absence of additional intervention. While this group allows for assessment of change over time relative to no treatment, it does not control for nonspecific factors such as attention, expectancy, or facilitator contact. Participants assigned to the WLC group will not receive any active intervention during the initial 4-week study period but will complete all study assessments on the same schedule as those in the treatment arms. Specifically, they will undergo baseline evaluation and complete identical outcome measures, such as self-reported self-transcendent state, acute pain ratings, EEG, and fNIRS, at time points matched to Sessions 1 and 4 of the intervention groups. They will also complete the full set of follow-up assessments at 1 week, 1 month, and 3 months postintervention to evaluate potential naturalistic changes in clinical outcome variables over time. Following the final assessment, participants in the waitlist group will be offered the opportunity to participate in a 2-hour MBI for chronic pain.

### Measures

Outcomes will be evaluated at two primary timescales: (1) during and immediately surrounding the mindfulness training sessions to assess mechanistic changes, including self-reported self-transcendence and psychophysiological responses, and (2) at baseline and follow-up intervals to examine the durability of clinical effects. Psychophysiological data (EEG and fNIRS), along with session-specific self-report measures, will be collected during the first and final training sessions (Sessions 1 and 4) to assess within-participant changes over the course of the intervention (see Table 2). Broader clinical outcomes, including pain interference, pain catastrophizing, and sleep disturbance, will be assessed remotely via online surveys at baseline and again at 1-week, 1-month, and 3-month postbaseline to evaluate the persistence of any treatment-related improvements (Table 3).

**Table 2.** State survey: overview of measures and corresponding measurement time points.

Measure	Target concept	Session 1	Session 2	Session 3	Session 4
NADA-S^a^	Self-transcendence	✓			✓
MPoD^b^	Decentering	✓			✓
Pain intensity (single item)	Pain intensity	✓			✓
EEG^c^	Frontal midline theta power	✓			✓
fNIRS^d^	Fronto-temporal-parietal cortical proxy of DMN^e^ activity; cerebral cortex	✓			✓

a NADA-S: Nondual Awareness Dimensional Assessment–State.

b MPoD: Metacognitive Processes of Decentering Scale.

c EEG: electroencephalogram.

d fNIRS: functional near-infrared spectroscopy.

e DMN: default mode network.

**Table 3.** Trait survey: overview of measures and corresponding measurement time points.

Measure	Target concept	T0^a^	T1^b^	T2^c^	T3^d^
BPI^e^ – Functional Interference	Functional Interference	✓	✓	✓	✓
NIH HEAL CDE^f^ Demographics	Demographic Information	✓			
BPI-PI^g^	Pain Intensity	✓	✓	✓	✓
Physical function	Physical function	✓	✓	✓	✓
PCS^h^	Pain catastrophizing	✓	✓	✓	✓
PHQ-2^i^	Depression	✓	✓	✓	✓
GAD-2^j^	Anxiety	✓	✓	✓	✓
PROMIS^k^ Sleep Disturbance – Short Form 6a	Sleep	✓	✓	✓	✓

a T0: 1-week pretreatment (Baseline).

b T1: 1-week post treatment.

c T2: 1-month post treatment.

d T3: 3-month post treatment.

e BPI: Brief Pain Inventory.

f NIH HEAL CDE: The Helping to End Addiction Long-term Initiative Common Data Elements.

g PI: Pain intensity

h PCS: Pain Catastrophizing Scale.

i PHQ-2: Patient Health Questionnaire 2-item.

j GAD-2: Generalized Anxiety Disorder 2-item.

k PROMIS: Patient-Reported Outcomes Measurement Information System.

### Primary Outcome

#### Primary Self-Report Outcome

The self-transcendent state will be assessed immediately before and after the first and final treatment sessions using the state version of the Nondual Awareness Dimensional Assessment (NADA-S). This validated 3-item measure captures momentary fluctuations in nondual awareness. Two items assess reductions in self-salience, and one assesses the presence of bliss. All items are rated on an 11-point numeric rating scale ranging from 0 (“not at all”) to 10 (“extremely”).

#### Primary Psychophysiological Outcomes

Frontal midline theta power will be measured by EEG. In Sessions 1 and 4, participants in the active treatment groups (MB and MB+DP) will complete psychophysiological data collection during a 5-minute resting baseline prior to treatment and throughout the 15-minute mindfulness practice. During the same sessions, participants in the WLC group will undergo a 5-minute resting baseline followed by a 15-minute time-matched control period. During all psychophysiological assessments, EEG will be continuously recorded from 10 midline scalp sites (Fz, F3, F4, FC1, FC2, FCz, Cz, CP1, CP2, and PZ) using an active sensor cap with Ag-AgCl electrodes (actiCap GmbH). In addition, vertical electrooculograms will be recorded. All recordings will be collected with an actiCHamp amplifier (Brain Products GmbH). Data will be acquired at a sampling rate of 500 Hz, a resolution of 0.489 µV and an amplification cutoff of 140 Hz, with impedances kept below 10 kilohms.

Connectivity in a validated fronto-temporal-parietal cortical proxy of DMN activity (ie, connectivity in-between channels belonging to the medial prefrontal cortex and the left and right MTG and STG, left and right posterior temporoparietal lobe, left and right temporoparietal junction will be measured by fNIRS during a 5-minute resting baseline period before treatment and during the 15-minute mindfulness practice. Hemodynamic responses to neuroactivation via oxy-, deoxy-, and total hemoglobin changes in the cerebral cortex will be measured with the NIRx NIRSport 2, a user-friendly, modular, and robust wireless fNIRS platform. The automated signal optimization algorithm ensures optimal signal quality before a measurement is started. Once data are being recorded, oxygenated hemoglobin and hemoglobin concentration changes can be visualized in real-time in several display modes. In addition, high-end whole-head visualizations are immediately available.

#### Acute Clinical Outcome

Acute pain intensity will be measured immediately before and after Sessions 1 and 4 using a single-item numeric rating scale: “How intense is your pain right now?” Responses are scored on an 11-point scale from 0 (“no pain”) to 10 (“worst pain imaginable”).

### Tertiary and Secondary Outcomes

#### Tertiary Clinical Outcomes

The following pain-related outcomes will be assessed using validated instruments from the Helping to End Addiction Long-term Initiative (NIH HEAL) Common Data Elements (CDE) battery at pretreatment and posttreatment time points (see Table 4): Brief Pain Inventory, Patient-Reported Outcomes Measurement Information System (PROMIS) Physical Functioning Short Form 6b, Brief Pain Catastrophizing Scale (PCS), Patient Health Questionnaire-2 (PHQ-2), Generalized Anxiety Disorder 2-Item Scale (GAD-2), and PROMIS Short Form v1.0–Sleep Disturbance 6a.

**Table 4.** Schedule of enrollment, interventions, and assessments (SPIRIT [Standard Protocol Items: Recommendations for Interventional Trials] diagram).

**Description**	**Study phase**			In-lab **procedures**				Follow-**u**p **p**ost**-t**reatment		
	Enrollment	Baseline	Allocation	Week 1	Week 2	Week 3	Week 4	1-week	1-month	3-month
**Time point**	–T1^a^	T0^b^						T1^c^	T2^d^	T3^e^
**Enrollment**										
Eligibility form	✓									
Informed consent	✓									
MINI^f^	✓									
**Randomization**										
Allocation			✓							
**Study arm**										
MB^g^				✓	✓	✓	✓	✓	✓	✓
MB+DP^h^				✓	✓	✓	✓	✓	✓	✓
WLC^i^				✓			✓	✓	✓	✓
**Assessments**										
NADA-S^j^				✓			✓			
EEG^k^				✓			✓			
fNIRS^l^				✓			✓			
MPoD^m^				✓			✓			
Acute pain intensity				✓			✓			
At-home mindfulness practice (MB and MB+DP only)				✓	✓	✓	✓			
BPI^n^		✓						✓	✓	✓
Physical function		✓						✓	✓	✓
PCS^o^		✓						✓	✓	✓
PHQ-2^p^		✓						✓	✓	✓
GAD-2^q^		✓						✓	✓	✓
PROMIS^r^ sleep disturbance		✓						✓	✓	✓
Pain catastrophizing		✓						✓	✓	✓

a –T1: Enrollment activities

b T0: 1-week pretreatment (baseline).

c T1: 1-week posttreatment.

d T2: 1-month posttreatment.

e T3: 3-month posttreatment.

f MINI: Mini-International Neuropsychiatric Interview.

g MB: mindful breathing.

h MB+DP: mindful breathing+direct pointing.

i WLC: waitlist control.

j NADA-S: Nondual Awareness Dimensional Assessment-State.

k EEG: electroencephalogram.

l fNIRS: functional near-infrared spectroscopy.

m MPoD: Metacognitive Processes of Decentering.

n BPI: Brief Pain Inventory.

o PCS: Pain Catastrophizing Scale.

p PHQ-2: Patient Health Questionnaire 2-item.

q GAD-2: Generalized Anxiety Disorder 2-item.

r PROMIS: Patient-Reported Outcomes Measurement Information System.

#### Pain

Participants will self-report their pain intensity, treatments for pain relief, effectiveness of those treatments, and pain interference with the 14-item BPI. Statements include “Please rate your pain by indicating the one number that best describes your pain at its WORST in the past 24 HOURS” with an 11-point Likert-like scale (0=no pain, 10=pain as bad as you can imagine). Five items measure recent and immediate pain intensity. One item asks participants about their pain treatments, and the next immediate item asks how much relief the treatments offered (from 0% to 100%). The next 7 items measure pain interference in the participant’s daily life with an 11-point Likert-like scale (0=does not interfere, 10=completely interferes).

#### Physical Function

Participants will self-report their physical function with the PROMIS Physical Function (PROMIS Physical Functioning Short Form 6b) questionnaire [45]. The PROMIS Physical Function questionnaire is a 6-item measure using a 5-point Likert scale (5=not at all, 1=cannot do) with questions such as “Does your health now limit you in doing two hours of physical labor?” Other questions, such as “Are you able to do chores such as vacuuming or yard work?,” use a 5-point Likert scale (5=without any difficulty, 1=unable to do).

#### Pain Catastrophizing

Participants will be asked about their pain catastrophizing with the Brief Pain Catastrophizing Scale (PCS) [36]. The Brief PCS is a validated, 5-item questionnaire on which participants rate their thoughts and feelings about pain on a 5-point Likert scale, ranging from 0 (“Not at all”) to 4 (“All the time”). Statements include “It’s awful, and I feel that it overwhelms me.”

#### Depression

Depressive symptoms were measured using the Patient Health Questionnaire-2 (PHQ-2), a 2-item questionnaire that serves as a brief screener for depression [35]. The PHQ-2 prompts patients to reflect on their mood over the previous 2 weeks, asking them how often they experienced “little interest or pleasure in doing things” or felt “down, depressed, or hopeless.” A 4-point Likert scale ranging from 0 (not at all) to 3 (nearly every day) is used to report the frequency of depressive symptoms, and a total score is determined from summing these points together. A total score of 3 or greater supports the need for further screening and indicates an increased likelihood of major depressive disorder.

#### Anxiety

Anxiety symptoms were measured using the Generalized Anxiety Disorder 2-Item Scale (GAD-2), a 2-item screener for generalized anxiety disorder [46]. Similar to the PHQ-2, the GAD-2 asks patients about how often they experienced certain symptoms over the past 2 weeks, including “feeling nervous, anxious, or on edge” and “not being able to stop or control worrying.” Both items are scored using a 4-point Likert scale that spans from 0 (not at all) to 3 (nearly every day). Points from these 2 items are added together to calculate a total score, with a total score of 3 or above being suggestive of generalized anxiety disorder.

#### Sleep

Sleep disturbance was assessed with the PROMIS Sleep Disturbance Scale (PROMIS Short Form v1.0–Sleep Disturbance 6a), a 6-item questionnaire that evaluates a patient’s overall sleep experience [33]. The first item evaluates a patient’s sleep quality over the past 7 days. Responses are scored on a 5-point Likert scale ranging from 1 (very poor) to 5 (very good). The other 5 items ask about the depth and restfulness of a patient’s sleep over the past 7 days. Sample items include “My sleep was refreshing” and “I tried hard to get to sleep.” As with the first item, these responses are also scored on a 5-point Likert scale ranging from 1 (not at all) to 5 (very much).

#### Process Measures

Engagement with at-home mindfulness practice will be monitored using a Qualtrics-based tracking system. Each participant will receive a unique survey link embedded with their participant ID to record the frequency and duration of practice. At the beginning of Sessions 2, 3, and 4, participants will also self-report their adherence to at-home exercises, including at-home mindfulness practice duration and any notable experiences.

As a manipulation check, state mindfulness will be assessed using the Metacognitive Processes of Decentering (MPoD) scale [47] administered immediately before and after Sessions 1 and 4. The MPoD captures momentary shifts in decentering, a key psychological mechanism thought to mediate mindfulness-related outcomes.

Participants will receive modest financial compensation at the baseline assessment and 3 follow-up time points. These compensation procedures are designed to recognize participant contributions while maintaining ethical standards for noncoercive remuneration.

### Statistical Analysis

#### Analysis Plan

The protocol will favor an intention-to-treat approach for the analysis of results. Data analysis will be performed in SPSS (IBM Corp). Baseline demographic information will be summarized using descriptive statistics. Continuous variables will be summarized with means and SDs. Categorical variables will be summarized with counts, proportions, and medians (IQR). The distribution of continuous variables will be assessed using graphical inspection and the Shapiro-Wilk test for normality. If the assumption of normality is not met, equivalent nonparametric approaches or data transformation will be used. The multiple testing problem will be accounted for using Bonferroni correction. All hypothesis tests will be 2-sided, and the familywise Type I error rate will be controlled at α=.05 using Bonferroni-adjusted significance thresholds. If needed, statistical analysis will also be conducted after controlling for baseline demographics and clinical characteristics (eg, age, sex, and race).

The effects of treatment on continuous variables will be evaluated using linear mixed models with fixed effects consisting of a time factor (before to after the experimental session; before treatment to 3-month follow-up) and a between-participants treatment factor (MB vs MB+DP vs WLC, as appropriate). If necessary, we will adjust for randomization imbalance by co-varying baseline variables. Little’s Missing Completely at Random (MCAR) test will be used to determine if data are missing completely at random. We will use maximum likelihood estimation procedures to deal with missing data according to an intent-to-treat philosophy that is robust against common patterns of missing data. The maximum likelihood estimation is based on all data observations; no values are deleted or imputed.

All EEG analyses will be performed in MATLAB (Matrix Laboratory; MathWorks) using scripts implementing the EEGLAB toolbox [48]. First, epochs of 300 s will be created for each participant. Then, the signal will be notch-filtered at 60 Hz and low-pass filtered at 40 Hz cutoff using a fourth-order Butterworth filter and high-pass filtered at 0.1 Hz cutoff using a second-order Butterworth filter. Filtered data will be passed through the PREP pipeline [49] which detrends the data, applies a notch filter tapering off the harmonics of 60 Hz, rereferences the data to the linked earlobe, and identifies and interpolates bad channels. Interpolation in low-density montages has been shown to produce valid data [49,50]. In the power spectral density analyses, average power spectra in the theta (4-8 Hz) band will be calculated using Welch periodogram across frontal midline sites.

All fNIRS data will be preprocessed in MATLAB as follows: detected NIR light will be transformed into O₂Hb and HHb concentrations using the modified Beer–Lambert law. Motion artifacts will be corrected using temporal derivative distribution repair, followed by band-pass filtering (0.01-0.1 Hz). Low-amplitude motion artifacts will be corrected using the algorithm of Cui et al [51], and single artifact-laden channels will be interpolated. A Gaussian PCA kernel will be applied to remove global signal components. Finally, signals will be converted to *z* scores to facilitate comparisons across participants.

### Plans to Promote Participant Retention and Complete Follow-Up

To promote participant retention and ensure complete follow-up, the study will implement a structured, participant-centered engagement strategy grounded in best practices for behavioral trial management. All follow-up assessments (at 1-week, 1-month, and 3-month posttreatment) will be conducted remotely via secure, web-based surveys to enhance flexibility and reduce burden. A dedicated RA will serve as each participant’s primary point of contact, maintaining regular communication and providing reminders and technical support based on participant preference (eg, phone, text, or email). Participants will receive modest compensation at baseline, each follow-up, and upon treatment completion, consistent with ethical standards for noncoercive remuneration.

Adherence will be further supported by weekly audio-guided mindfulness recordings and at-home practice diaries. Flexible scheduling will be offered for in-person EEG and fNIRS assessments to accommodate work and caregiving responsibilities. Missed sessions or assessments will be followed up within 48 hours, with up to 3 recontact attempts using alternate methods. Participants may re-engage at the next appropriate activity without penalty and may request a personalized summary of their self-report data poststudy. This approach aims to promote retention and data completeness while minimizing participant burden.

### Ethical Considerations

The Florida State University IRB approved all study procedures prior to participant enrollment (STUDY00004506). All procedures comply with institutional and international ethical standards. AEs will be monitored and reported per IRB policy. This study was prospectively registered on Open Science Framework (OSF): DOI 10.17605/OSF.IO/3UY2C. Trial results will be disseminated via peer-reviewed publications, academic conferences, and stakeholder engagement efforts. A summary of findings will also be offered to participants following study completion.

All participants provided informed consent prior to the participation in the study, in accordance with institutional guidelines and the 1964 Declaration of Helsinki. Any publications resulting from this research will not contain any identifiable information in any form. A model consent form will be made available from the corresponding author on reasonable requests to qualified individuals.

After eligibility has been established, individuals who express interest in participating in the study will complete the informed consent process under the guidance of a trained RA, in accordance with IRB requirements and ethical research standards. Participants will have the option to receive a copy of the signed consent form via email for their records. All consent documentation will be stored securely in compliance with institutional data protection policies.

Data will be deidentified and stored on secure servers at CHIL. Any paper data (questionnaires, sociodemographic, and clinical info) will have identifiers removed and banked for a minimum of 3 years. Deidentified data will be available to the research team, including the PI (AWH), the research coordinator, and the RAs. Any electronic data will be stored on a password-protected computer. Computers will be stored in locked rooms. Any data collected online will be stored on an encrypted data server at the College of Nursing. Any downloaded data will be stored on a password-protected computer. The RA supervised by the PI will be responsible for data receipt. AWH will be responsible for data analysis and results interpretation.

## Results

At the time of the initial manuscript submission, this protocol is Version 4, dated May 20, 2025. Recruitment for the trial began on October 28, 2024, is ongoing, and is expected to be completed by May 2027. Final results will be reported in accordance with CONSORT (Consolidated Standards of Reporting Trials) guidelines and submitted for peer-reviewed publication. Any substantial protocol amendments will be documented with updated version numbers and dates and communicated to relevant regulatory bodies, investigators, and trial registries as required.

## Discussion

CMP continues to pose a significant public health burden, in part due to its capacity to become enmeshed with the sense of self. The present randomized controlled clinical trial is designed to move beyond symptom reduction alone by examining whether altering the self-referential structure of pain perception, via DP instruction, can produce meaningful and durable analgesic effects. DP may offer relief by shifting how individuals relate to pain, reducing identification with painful sensations and loosening the pain-laden self-concept that can sustain the pain they endure. The Pointing Beyond project is designed to make 4 key contributions to the field of mindfulness-based pain relief.

The first contribution concerns measurement. By combining momentary self-report with concurrent psychophysiological indices, frontal midline theta activity via EEG and DMN dynamics via fNIRS, this trial moves beyond the retrospective and aggregate outcome data that have characterized most prior work on self-transcendence in mindfulness research. Real-time triangulation of subjective experience and neural activity allows for a more precise characterization of when and how self-transcendent states arise during practice and whether their phenomenological and neurobiological signatures are coherently coupled. This multimodal approach is essential because self-report measures of transcendence are inherently subjective and susceptible to demand characteristics; convergence with independent psychophysiological indices would substantially strengthen causal inference about the mechanism.

Second, a central contribution is the dismantling design itself. Comparing MB alone against MB+DP against a waitlist control isolates the specific contribution of direct pointing instruction to both self-transcendence and analgesia. Prior trials incorporating DP, including RCTs of the MORE program and brief perioperative MBIs, confounded DP with other intervention components, leaving its independent effect unexamined. If MB+DP produces meaningfully greater self-transcendence and pain relief than MB alone, this would provide the first direct evidence that DP adds analgesic value beyond standard mindfulness instruction. This finding would have immediate implications for how brief MBIs are designed and delivered in clinical settings.

Further, this trial advances the goals of personalized medicine by investigating whether different mindfulness practice instructions produce distinct phenomenological experiences and clinical outcomes. Comparing MB and MB+DP at the level of both phenomenological experience and neural activity advances the goals of mechanism-based treatment personalization. If the two conditions diverge in their effects on self-transcendence but converge on pain outcomes, this would suggest that multiple pathways can produce equivalent clinical benefit and that individual preference and acceptability, rather than specific instructional content, should guide practice selection. Conversely, if MB+DP produces superior outcomes on both mechanistic and clinical indices, this would support a more targeted prescriptive approach, particularly for patients whose pain experience is strongly identity-bound. Either pattern of results would meaningfully inform clinical decision-making in a field that currently lacks guidance on how to match mindfulness instruction style to patient presentation.

The fourth contribution concerns scalability. Demonstrating that just four 30-minute sessions can produce durable reductions in pain interference through three-month follow-up would represent a meaningful addition to the feasibility literature on brief MBIs. In contrast to multiweek programs like MBSR or MORE, which impose substantial time and access burdens, this trial evaluates the effects of just 2 hours of in-person training (across 4 30-minute sessions) paired with 15 minutes of daily practice. An minfulness intervention of this brevity could realistically be integrated into primary care, rehabilitation settings, or digital health platforms. Short, scalable interventions are especially vital for reaching populations with limited access to traditional programs, such as rural residents or those with time, mobility, or financial constraints.

Several challenges warrant consideration. Capturing transient self-transcendent states during brief interventions requires sensitive instrumentation and careful interpretation. While fNIRS offers valuable ecological validity, its spatial resolution limits assessment of deeper DMN structures such as the posterior cingulate cortex. Moreover, ensuring fidelity in delivering nuanced practices such as DP necessitates skilled facilitators and robust supervision protocols. Despite these limitations, the study’s emphasis on real-time neural and experiential assessment in naturalistic settings enhances its translational relevance.

The Pointing Beyond project represents a theoretically grounded and methodologically rigorous effort to identify the specific mechanisms through which mindfulness produces pain relief. By isolating the contribution of direct pointing instruction, triangulating subjective and neurobiological indices of self-transcendence, and testing their mediating role in both acute and sustained pain outcomes, this trial directly contributes to the scientific understanding of the experiential and neural processes through which mindfulness relieves CMP.

## Supplementary material

10.2196/82362 Checklist 1 SPIRIT (Standard Protocol Items: Recommendations for Interventional Trials) checklist.

## Abbreviations

AE: adverse event
BPI: Brief Pain Inventory
CDE: Common Data Elements
CHIL: Complementary Health Innovation Lab
CMP: Chronic musculoskeletal pain
CON: College of Nursing
DMN: Default mode network
DP: direct pointing
EEG: Electroencephalography
fNIRS: functional near-infrared spectroscopy
FSU: Florida State University
GAD-2: Generalized Anxiety Disorder 2-item
IRB: Institutional Review Board
ITT: Intention-to-treat
MAR: Missing at random
MATLAB: Matrix Laboratory
MB: mindful breathing
MBI: Mindfulness-based interventions
MICE: Multiple imputations by chained equations
MINI: Mini-International Neuropsychiatric Interview
MORE: Mindfulness-Oriented Recovery Enhancement
MPoD: Metacognitive Processes of Decentering Scale
NADA-S: Nondual Awareness Dimensional Assessment – State
NIH HEAL: The Helping to End Addiction Long-term Initiative
NMAR: not missing at random
PCS: Pain Catastrophizing Scale
PHQ-2: Patient Health Questionnaire 2-item
PI: principal investigator
PROMIS: Patient-Reported Outcomes Measurement Information System
RA: research assistant
RCT: Randomized controlled trial
SPIRIT: Standard Protocol Items: Recommendations for Interventional Trials
WLC: waitlist control
